# De lessen die de zorg kán en móét leren van de COVID-19-pandemie

**DOI:** 10.1007/s12508-020-00268-6

**Published:** 2020-07-16

**Authors:** Daan Westra, Maike Tietschert

**Affiliations:** 1 faculteit Health, Medicine and Life Sciences, CAPHRI, vakgroep Gezondheidszorgonderzoek, Universiteit Maastricht, Maastricht, Nederland; 2 faculteit der Sociale Wetenschappen, vakgroep Organisatiewetenschappen, Vrije Universiteit, Amsterdam, Nederland; 3 Clinical Excellence Center, Stanford Medical School, Palo Alto, CA Verenigde Staten

**Keywords:** management van zorg, COVID-19, inzetbaarheid, samenwerken, e‑health, Healthcare management, COVID-19, Employability, Collaboration, E‑health

## Abstract

De COVID-19-pandemie stelt zorgorganisaties voor grote uitdagingen. Hoewel specifieke aspecten prominenter zijn in bepaalde sectoren van de zorg, staan alle zorgorganisaties voor de opgave om de randvoorwaarden te organiseren waarbinnen zorgprofessionals zo veel mogelijk mensen zo goed mogelijk kunnen helpen. Vanuit managementperspectief vallen vier grote thema’s op in de manier waarop zorgorganisaties hiermee omgaan. Op de eerste plaats proberen zorgorganisaties met man en macht hun personeel inzetbaar te houden. Daarnaast worden er verschillende digitale oplossingen geïmplementeerd en wordt er op ongekende schaal en snelheid samengewerkt met organisaties binnen en buiten de zorg. Dit gebeurt in directe interactie met landelijk en regionaal beleid, dat als last of lust ervaren wordt. Om goed door de coronacrisis te komen en de zorg op een duurzame manier te organiseren in het ‘nieuwe normaal’ na de crisis, is het essentieel dat de lessen en best-practices van dergelijke initiatieven systematisch getrokken en verspreid worden.

## Abstract (en)

The COVID-19 pandemic presents healthcare organizations with substantial challenges. Four main themes can be distinguished in the way healthcare organizations organize the necessary preconditions for their professionals to provide high quality care in this crisis. These include: keeping professionals employable, implementing a wide range of digital solutions, collaborating with organizations in and outside of healthcare at a large scale and rapid pace, and interacting with national and regional policy, which can either serve as a barrier or facilitator. It is essential to learn from these approaches and rapidly disseminate the associated best practices.

## Inleiding

De COVID-19-pandemie stelt zorgorganisaties voor substantiële uitdagingen [1]. In verschillende sectoren worstelen organisaties met specifieke vraagstukken. Zo richten ziekenhuizen zich vooral op het vergroten van de intensive care-capaciteit, trachten verpleeghuizen zonder bezoek toch met compassie zorg te verlenen, moeten ggz-aanbieders en huisartsen vooral op afstand zorg leveren, proberen jeugdzorgaanbieders jongeren in kwetsbare situaties zo goed mogelijk te begeleiden en is het voor professionals in contactberoepen, zoals tandartsen en fysiotherapeuten, überhaupt moeilijk om zorg te leveren. Hoewel zorgorganisaties in verschillende sectoren elk aan specifieke randvoorwaarden moeten voldoen, staan ze wel degelijk voor vergelijkbare uitdagingen. Ook in ziekenhuizen en bij huisartsenpraktijken kan veel niet-acute zorg niet geleverd worden. De Nederlandse Zorgautoriteit rapporteerde dat er gedurende de eerste tien weken van de coronacrisis bijna 700.000 verwijzingen van huisartsen naar tweedelijnsinstellingen zijn uitgebleven en liet bij monde van voorzitter Marian Kaljouw weten dat het wegwerken van dit ‘stuwmeer’ aan zorg een ‘kwestie van heel lange adem’ is [2, 3]. Adviesbureau Gupta Strategists becijferd zelfs dat deze uitgestelde zorg meer gezonde levensjaren heeft gekost dan dat de zorg voor coronapatiënten heeft opgeleverd [4]. Dát er voor zorgorganisaties veel is veranderd staat vast. Zij worstelen daardoor allemaal met vragen als: hoe houden we onze medewerkers inzetbaar, hoe implementeren we snel en effectief digitale oplossingen, hoe kunnen we zo goed mogelijk samenwerken met andere organisaties en ons zorgaanbod optimaal op elkaar afstemmen en hoe gaan we om met de onzekerheid van constant veranderende nationale en regionale beleidskaders?

Er bestaat uiteraard geen eenduidig antwoord op deze vragen. Hoewel over de hele wereld gezocht wordt naar manieren om de coronacrisis te beheersen, biedt het onderzoek tot op heden verrassend weinig aanknopingspunten op deze vragen. Het huidige COVID-19-gerelateerde onderzoek valt grofweg in een aantal stromingen te verdelen [5]. Logischerwijs is er ten eerste het medische onderzoek, dat erop gericht is om de nieuwe ziekte en bijbehorende symptomen te begrijpen, risicofactoren aan te wijzen en effectieve behandelingen te ontwikkelen. Vooraanstaande medische tijdschriften stellen deze kennis veelal gratis ter beschikking. De tweede stroom, het epidemiologische onderzoek, is in deze tijd van crisis minstens zo belangrijk en richt zich primair op het verklaren en voorspellen van besmettingspatronen binnen (specifieke) populaties en maatregelen om de verspreiding van het virus af te remmen. Een derde stroom aan onderzoek is voornamelijk beschrijvend van aard en richt zich op de verschillende beleidsmaatregelen die landen nemen in hun strijd tegen de crisis. De OECD en Wereldgezondheidsorganisatie houden hier bijvoorbeeld overzichten van bij [6]. Het huidige onderzoek helpt medici om COVID-19-patiënten zo effectief mogelijk te behandelen. Het brengt voor beleidsmakers de urgentie van het probleem in kaart en geeft hen beleidsinstrumenten om het probleem aan te pakken. Het belang van beide kan niet onderschat worden, zo wordt ook door premier Rutte veelvuldig onderschreven. Bestuurders en managers van zorgorganisaties hebben echter veel minder houvast aan het huidige onderzoek en daardoor behoefte aan andersoortige inzichten [1, 5].

De primaire waarde van management binnen zorgorganisaties zit in het scheppen van de randvoorwaarden waarbinnen zorgprofessionals zo veel mogelijk patiënten zo goed mogelijk kunnen helpen, zeker in tijden van crisis. In landelijke en lokale media verschijnen dagelijks berichten van een heel scala aan initiatieven en oplossingen die zorgorganisaties in dat kader gebruiken. Afgezien van het vergroten van de intensive care-capaciteit in ziekenhuizen en pogingen om de aanlevering van cruciale middelen, zoals beschermend materiaal, te garanderen, vallen vanuit management- en organisatieperspectief vier overkoepelende thema’s op in deze berichtgeving. Gezien het onzekere beloop van de crisis en de kans op een nieuwe golf coronapatiënten of een volgende pandemie, is het niet alleen van groot belang om de bestaande kennis rondom deze thema’s te benutten, maar ook om de kennis tegen het licht van de crisis te verdiepen en zorgorganisaties te voorzien van de bijbehorende lessen en best-practices.

## Medewerkers op één

Op de eerste plaats hebben zorgorganisaties meer dan ooit tevoren zorgen over en aandacht voor de inzetbaarheid van hun medewerkers. Zonder inzetbare professionals hebben andere maatregelen per definitie geen effect. Onder normale omstandigheden is de druk op zorgprofessionals al hoog en zijn burn-outpercentages en de gevolgen daarvan voor professionals en patiënten groot [7, 8]. Het werken in de huidige crisissituatie vergt logischerwijs extra veel van ze. Zorgorganisaties moeten daarbij op de eerste plaats de fysieke gezondheid van hun medewerkers garanderen, bijvoorbeeld in de vorm van voldoende beschermend materiaal dat ervoor zorgt dat medewerkers het virus zelf niet oplopen. Lange diensten, veel (onverwachte) sterfgevallen, ingetrokken vakantiedagen, andere dan de normale werkzaamheden (soms op andere afdelingen of binnen andere organisaties), de lastige combinatie van werk en privé, en de onzekerheid over hoe lang dit nog duurt vergen daarnaast ook ongekende draagkracht van zorgprofessionals. Hoewel de maatschappij voor zorgverleners klapt, is het primair aan zorgorganisaties zelf om in deze omstandigheden goed voor hun personeel te zorgen [9]. Managementonderzoek suggereert dat organisaties niet alleen burn-outsymptomen bij professionals moeten bestrijden, maar zich vooral op een manier moeten organiseren die de kans hierop zo klein mogelijk maakt. Bijvoorbeeld door sensitief te zijn voor de emoties van hun personeel, regelmatig check-in’s te organiseren, emotionele support voor medewerkers beschikbaar te stellen en de nadruk juist niet op prestatienormen te leggen [1]. Het bepalen, begrijpen en verspreiden van best-practices om goed voor zorgpersoneel te zorgen is niet alleen in tijden van crisis van onschatbare waarde, maar kan tot lang daarna zijn vruchten afwerpen.

## Digitale dienstverlening

Ten tweede schakelen veel zorgorganisaties in rap tempo over op digitale dienstverlening. Karify, een platform dat videosessies in de ggz faciliteert, zag eind maart het aantal sessies elke dag verdubbelen [10]. Performation becijferde dat ziekenhuispoli’s vijfmaal zo vaak gebruikmaken van beeldbellen [11]. Ook in het buitenland implementeren zorgorganisaties massaal digitale diensten. Waar voor de coronacrisis wereldwijd bedenkingen waren rond het gebruik van digitale oplossingen, is het momenteel vaak de enige manier om überhaupt nog een vorm van zorg te kunnen leveren [12]. In Schotland wordt zelfs een verhoging van 1.000% gerapporteerd in het gebruik van beeldbellen [12]. De razendsnelle adoptie van digitale oplossingen doet echter niet alleen de privacy-technische vragen herleven, het stelt zorgorganisaties ook voor praktische keuzen voor specifieke oplossingen en softwareleveranciers [12]. Daarnaast rijzen er ook fundamentelere vragen over de integratie en afstemming van digitale diensten met het overige zorgaanbod, de kwaliteit van de geleverde zorg en (veranderingen in) de perceptie van patiënten en zorgprofessionals rond digitale diensten.

## Samenwerking

Op de derde plaats hebben zorgorganisaties de handen massaal en adequaat ineengeslagen. Op verschillende plekken in het land zijn coronacentra opgezet, die bemenst worden door medewerkers van verschillende zorgaanbieders, en patiënten van overbezette ziekenhuizen worden overgeplaatst naar ziekenhuizen in andere delen van het land of zelfs naar ziekenhuizen in Duitsland [13, 14]. Ook met private partijen en burgerinitiatieven buiten de zorg wordt op ongekende schaal en in een hoog tempo samengewerkt. Hotelketens stellen locaties ter beschikking, horecaondernemingen voorzien zorgorganisaties van eten, en via verschillende platformen bieden mensen aan om in ziekenhuizen te werken of op kinderen van zorgprofessionals te passen. Dergelijke prestaties zijn simpelweg verbluffend. Onder normale omstandigheden komen samenwerkingsverbanden in de zorg over het algemeen moeizaam tot stand en duurt het drie tot vijf jaar voordat dergelijke samenwerkingsverbanden hun vruchten afwerpen [15, 16]. Het is daarom evident dat we niet alleen moeten leren hoe we deze samenwerkingsverbanden in stand kunnen houden, maar ook hoe we vergelijkbare vormen van samenwerking kunnen initiëren. Eens te meer omdat, onder het motto van de juiste zorg op de juiste plek, regionale samenwerking voor de crisis een van dé speerpunten was om de zorg voor de toekomst duurzaam te houden [17].

## Dynamische context

Tot slot dienen de aanpassingen die zorgorganisaties gedurende de crisis doorvoeren afgestemd te zijn op omgevingsfactoren die voor elke organisatie anders zijn en die bovendien snel veranderen. Zo is het aantal coronabesmettingen bijvoorbeeld niet evenredig over het land verdeeld [18], waardoor de urgentie voor drastische veranderingen per organisatie en regio kan verschillen. Anderzijds veranderen de beleidskaders waarbinnen zorgorganisaties moeten en kunnen handelen terwijl de situatie zich ontvouwt. Zo was het voor verpleeghuizen een tijd niet toegestaan om bezoek toe te laten, mogen bewoners inmiddels één vaste bezoeker ontvangen en wordt er al gesproken over het verruimen van de regel op korte termijn [19]. De snelle aanpassingen die zorgorganisaties hebben gemaakt zijn, gezien het sterk geïnstitutionaliseerde karakter van de zorgsector, bewonderenswaardig te noemen [20]. Al met al dwingen specifieke omgevingsfactoren zorgorganisaties ertoe om voortdurend af te wegen of ze een specifieke situatie zelf kunnen oplossen en of ze dat ook daadwerkelijk mogen. Voor een aantal majeure thema’s, zoals de beschikbaarheid van beschermend materiaal, de verdeling van ic-capaciteit en de bevoorschotting door inkopende partijen, zijn bijvoorbeeld landelijke oplossingen gevonden. Andere vraagstukken, zoals de zorg voor coronapatiënten buiten het ziekenhuis, zijn vooral lokaal opgelost [14]. Gedurende de evaluatie van deze crisis en de terugkeer naar het nieuwe normaal is het daarom van belang om te doorgronden welke mate van beleidsvrijheid organisaties in staat stelt om hun creatieve vermogen maximaal te benutten en de voorgaande drie randvoorwaarden te creëren.

## Blik vooruit

Zorgorganisaties zoeken naar manieren om hun medewerkers, ondanks de uitzonderlijke omstandigheden, in staat te stellen hun patiënten zo goed mogelijk van dienst te blijven zijn. De vraagstukken die daarmee gepaard gaan worden door vooraanstaande organisatiewetenschappers niet voor niets gelijkgesteld aan de mate van de medische uitdagingen die COVID-19 met zich meebrengt [1]. Begrijpen hoe zorgorganisaties met de crisis zijn omgegaan, welke reacties goed werken en waarom dat zo is, en de geleerde lessen breed delen in de sector geven zorgorganisaties enerzijds houvast om door onzekere tijden te blijven navigeren. Daarmee heeft het rechtstreeks gevolgen voor de manier waarop we als maatschappij door de crisis komen. In een periode waarin de meeste zorgorganisaties beginnen met het opstarten van hun uitgestelde zorg moeten we ons terdege afvragen of, en zo ja op welke manier, de crisis zorgorganisaties niet voorgoed heeft veranderd. Zelfs wanneer medici effectieve behandelingen tegen de nieuwe ziekte in handen hebben, het gevaar van nieuwe besmettingshaarden epidemiologen niet meer 24/7 wakker doet liggen en beleidsmakers de laatste coronamaatregel hebben opgeheven rest voor zorgorganisaties nog altijd de vraag ‘hoe nu verder?’. Het wordt tijd dat we dat, bijvoorbeeld binnen het COVID-19-programma van ZonMw, grondig onderzoeken.
